# Deep Phenotypic Characterisation of CTCs by Combination of Microfluidic Isolation (IsoFlux) and Imaging Flow Cytometry (ImageStream)

**DOI:** 10.3390/cancers13246386

**Published:** 2021-12-20

**Authors:** Antonio J. Ruiz-Rodríguez, Maria P. Molina-Vallejo, Inés Aznar-Peralta, Cristina González Puga, Inés Cañas García, Encarna González, Jose A. Lorente, M. Jose Serrano, M. Carmen Garrido-Navas

**Affiliations:** 1 Clinical Management Unit of Digestive Disease, San Cecilio University Hospital, 18016 Granada, Spain; antjosruirod@outlook.es; 2 GENYO Centre for Genomics and Oncological Research: Pfizer, University of Granada, Andalusian Regional Government, Liquid Biopsy and Cancer Interception Group, PTS Granada, 18016 Granada, Spain; maria.molina@genyo.es (M.P.M.-V.); ines.aznar@genyo.es (I.A.-P.); jose.lorente@genyo.es (J.A.L.); 3 Legal Medicine Department, Medicine School, University of Granada, 18016 Granada, Spain; 4 Clinical Management Unit of Surgery, San Cecilio University Hospital, 18016 Granada, Spain; crisgona2@hotmail.com (C.G.P.); inescanasgarcia@gmail.com (I.C.G.); 5 Clinical Management Unit of Oncology, University Hospital Virgen de las Nieves, 18014 Granada, Spain; encarnagonzalezflores@gmail.com; 6 Medical Oncology Department, Bio-Health Research Institute (IBS, Granada), University Hospital Virgen de las Nieves, University of Granada, 18012 Granada, Spain; 7 Department of Pathological Anatomy, Faculty of Medicine, Campus de Ciencias de la Salud, University of Granada, 18016 Granada, Spain; 8 Genetics Department, Faculty of Sciences, University of Granada, 18071 Granada, Spain

**Keywords:** circulating tumour cells, IsoFlux, ImageStream, CRC, CTC heterogeneity

## Abstract

**Simple Summary:**

Cells that escape the primary tumour and have the potential ability to colonise distant organs through metastasis are called circulating tumour cells (CTCs). The study of CTCs in colorectal cancer (CRC) has demonstrated their prognostic utility, although current methodologies only allow the evaluation of CTC numbers and a maximum of two markers. Here, we developed a novel protocol for the isolation and characterisation of CTCs by combining two existing technologies. This new methodology allows the simultaneous evaluation of multiple markers and parameters. In particular, we evaluated the expression of a mutant protein (BRAF^V600E^) associated with poor response to therapies against EGFR and the expression of PD-L1, a marker for immunotherapy. Based on these markers, we evaluated the CTCs (positive for cytokeratin) of 16 early CRC patients and demonstrated the suitability of our protocol to classify patients into potential responders and non-responders.

**Abstract:**

The isolation of circulating tumour cells (CTCs) in colorectal cancer (CRC) mostly relies on the expression of epithelial markers such as EpCAM, and phenotypic characterisation is usually performed under fluorescence microscopy with only one or two additional markers. This limits the ability to detect different CTC subpopulations based on multiple markers. The aim of this work was to develop a novel protocol combining two platforms (IsoFlux^TM^ and ImageStream^®X^) to improve CTC evaluation. Cancer cell lines and peripheral blood from healthy donors were used to evaluate the efficiency of each platform independently and in combination. Peripheral blood was extracted from 16 early CRC patients (before loco-regional surgery) to demonstrate the suitability of the protocol for CTC assessment. Additionally, peripheral blood was extracted from nine patients one month after surgery to validate the utility of our protocol for identifying CTC subpopulation changes over time. Results: Our protocol had a mean recovery efficiency of 69.5% and a limit of detection of at least four cells per millilitre. We developed an analysis method to reduce noise from magnetic beads used for CTC isolation. CTCs were isolated from CRC patients with a median of 37 CTCs (IQ 13.0–85.5) at baseline. CTCs from CRC patients were significantly (*p* < 0.0001) larger than cytokeratin (CK)-negative cells, and patients were stratified into two groups based on BRAF^V600E^ and PD-L1 expression on CK-positive cells. The changes observed over time included not only the number of CTCs but also their distribution into four different subpopulations defined according to BRAF^V600E^ and PD-L1 positivity. We developed a novel protocol for semi-automatic CTC isolation and phenotypic characterisation by combining two platforms. Assessment of CTCs from early CRC patients using our protocol allowed the identification of two clusters of patients with changing phenotypes over time.

## 1. Introduction

Circulating tumour cells (CTCs) are one of the most widely studied types of liquid biopsy and have been used as prognostic and monitoring biomarkers [1] in most solid tumours, such as colorectal [2], lung [3] and breast [4] cancers. Evaluation of CTCs requires two essential steps: enrichment (or isolation) and enumeration (with or without characterisation). Several protocols and platforms have been developed for their isolation, with two distinct approaches: label-free strategies, including the use of red blood cell (RBC) lysis buffers, size selection (ISET, Rarecells, Inc. USA), Parsortix (Angle plc, CA, USA), CanPatrol (SurExam, China), density gradient centrifugation, etc., or label-dependent strategies, including negative selection (mainly using CD45 exclusion) or positive selection with a variety of markers depending on the tumour type (EpCAM being the most widely used).

The IsoFlux^TM^ system (Fluxion Biosciences, Inc., Alameda, CA, USA) is a semi-automatic platform for the enrichment of CTCs using a combination of immunomagnetic positive selection and microfluidics, and it significantly increases isolation efficiencies compared to the FDA-approved CellSearch^®^ (Menarini, Italy) system [5]. An improvement in the recovery rate from 40% to 90% using cancer cell lines was achieved compared with CellSearch^®^ [6], and this technology was used to successfully isolate CTCs in bladder [7], prostate [8], ovarian [9], liver [10,11,12,13] and pancreatic [14] cancer patients. However, it was only recently that the IsoFlux^TM^ (Fluxion, Biosciences, Inc., Alameda, CA, USA) platform was used to isolate CTCs from colorectal cancer patients to evaluate the relationship between microsatellite instability (MSI) status and peri-operative release of CTCs [15]. In any case, enumeration of CTCs isolated with this platform is always performed by using immunocytochemistry (mainly looking for cytokeratin positivity and CD45 negativity). The use of fluorescent/confocal microscopy not only involves manual characterisation/enumeration of CTCs but also limits the number of biomarkers that can be analysed simultaneously.

The Amnis^®^ ImageStream^®X^ MKII (Luminex, Belgium), ISx, flow cytometer has 12 detection channels divided into two CCD cameras with 6 channels, each allowing the simultaneous evaluation of up to 12 markers [16] and including a microscopical picture of each event that is being interrogated. This system was used after manual enrichment using EpCAM microbeads (Miltenyi Biotec, Germany) to phenotypically characterise CTCs and compared with the standard CellSearch^®^ (Menarini, Italy) CTC isolation technology, and there were no significant differences in the detection of CTCs between the two technologies [17]. Later, CTCs from hepatocellular carcinoma patients were detected by the ISx using prior CD45 depletion after red blood cell lysis [18], and the results showed that CTCs isolated from 65% of the patients were correlated with poorer survival. Following the same protocol, CTCs isolated from oesophageal, thyroid and ovarian cancer patients were also characterised using the ISx [19], and the results demonstrated the potential of this technology for multi-parametric phenotypic characterisation. Recently, a recovery rate of 40% with a limit of detection of 5 cells in 7.5 mL of blood was achieved using the ISx after a previous enrichment protocol based on size selection [20], and the ISx was even able to identify CTCs in neuroblastoma patients without a prior enrichment protocol (only red blood cell lysis) [21]. However, to date, no effort has been made to combine IsoFlux^TM^ (Fluxion, Biosciences, Inc., Alameda, CA, USA) for CTC isolation and ISx for CTC characterisation.

Low levels of CTCs are identified in pre-tumoural diseases such as colorectal polyps, and in colorectal cancer, the number of CTCs is associated with the tumour site, being greater in patients with sigmoid colon tumours compared with other locations [22]. Pre-operative CTC numbers in CRC patients subjected to surgery were associated with poorer overall survival [23,24,25], and it was shown to have diagnostic utility in early disease stages, as the number of CTCs was significantly greater in colorectal carcinoma compared with colorectal polyps patients [22]. However, most studies on CTCs have relied solely on the expression of epithelial markers (EpCAM/CK) versus the absence of CD45 expression [12,24], reducing the ability to detect/characterise CTCs and thus weakening their prognostic value [26]. Furthermore, poor prognosis is associated not only with CTC counts but also with CTC clusters (also called microemboli), as they increase with disease stage and are significantly associated with worse outcomes [27].

Somatic mutations in *BRAF* are found in 10–15% of metastatic colorectal cancer (mCRC) patients, with *BRAF^V600E^* being found in most of them (95%). The presence of this variant is considered a poor prognostic factor with a median overall survival below 20 months in mCRC [28]. Patients with this mutation and microsatellite-stable (MSS) tumours have a poorer prognosis, whereas, for those with microsatellite instability (MSI), this mutation is associated with sporadic carcinoma and a better prognosis [29]. In fact, *BRAF*-driven tumours follow a different path compared to the “classical adenoma-carcinoma” hypothesis and are associated with sessile serrated adenomas [30]. BRAF is a downstream effector of the EGFR pathway, and when mutated, it favours cell proliferation and survival. The efficiency of anti-EGFR depends on the *KRAS/BRAF* mutation status, as it was demonstrated that *BRAF*-mutated tumours had reduced response rates to anti-EGFR, even though they were *KRAS* wild-type [31]. Beyond anti-EGFR treatments, patients with *BRAF^V600E^* tumours were treated with immunotherapy for anti-PD-L1 in the CheckMate 142 clinical trial [32], as it was shown that PD-L1 is upregulated by EGFR activation. Results from this clinical trial suggest that *BRAF^V600E^* may regulate PD-L1 expression [33], although other clinical trials showed that the presence of the *BRAF^V600E^* mutation is not a predictor of anti-PD-1 treatment response [29], suggesting that they might act independently. In fact, a combination of anti-PD-L1 and anti-BRAF treatments is suggested to be an interesting approach [34]. Although CRC is considered a cold tumour for PD-L1 expression, which is only present in 25% of patients [35], its expression has been associated with disease progression, particularly in early CRC [36]. In fact, not only the expression levels but also the localisation of PD-L1 are considered a prognostic factor, as nuclear PD-L1 staining in vimentin-positive CTCs has been significantly associated with worse overall survival [37].

Here, we optimised a combined protocol in which CTC isolation is performed by the IsoFlux^TM^ (Fluxion, Biosciences, Inc., Alameda, CA, USA) technology, followed by phenotypic characterisation using the ImageStream^®X^ (Luminex, Belgium) platform for the joint evaluation of BRAF^V600E^ and PD-L1 expression on CTCs from early-stage colorectal cancer patients.

## 2. Materials and Methods

### 2.1. Protocol Optimisation Experiments

For the control experiments, the human colorectal cancer cell lines HT-29 and SW480 (BRAF^V600E^-positive and -negative, respectively) were cultured using Dulbecco’s Modified Eagle Medium (DMEM) + 10% FBS. Cells were assessed for mycoplasma contamination, and cell authenticity was confirmed by STR. Human PBMCs from five healthy donors were also used in spike-in controls; these cells were positive for the anti-CD45 antibody and negative for the remaining tumour-specific antibodies.

IsoFlux recovery rate experiments: To evaluate the efficiency of CTC enrichment, dilutions of 300 cultured cells (HT-29 and SW480) in PBS were run in duplicate on the IsoFlux (Fluxion, Biosciences, Inc., Alameda, CA, USA). To ensure minimum error for cell counts, we diluted 2–3 million cells from tissue culture (in 3 mL of 1× PBS) at 1/100, and then 5 µL of the dilution was visually counted under the microscope (as this volume covered the visual diameter of the 20× objective). Then, the appropriate volume from the 1/100 dilution was added to a total volume of 500μL of PBS. After the IsoFlux^TM^ run, the paraformaldehyde-fixed recovered cells were manually counted on a fluorescence microscope for cytokeratin staining (anti-CK-FITC, 130-060-301, Miltenyi Biotec, Germany), and the ratio of cell recovery was calculated for each cell line.

Compensation matrix for antibody staining: UltraComp eBeads™ (Thermo Fisher, MA, USA) were used following the manufacturer’s protocol to adjust the laser intensities of the ImageStream^®X^ (Luminex, Belgium) for each antibody (see Table 1) using control cell lines and PBMCs from healthy donors to design a compensation matrix and to reduce false-positive signals from cellular autofluorescence.

Single-stained controls: Culture cell lines as well as PBMCs were fixed and permeabilised using 1.7% paraformaldehyde and 0.2% Triton. Then, antibody labelling for cytokeratin (CK), BRAF^V600E^, PD-L1 and CD45 was performed using the antibodies in Table 1 and 7-AAD (for nuclear staining) for 20 min at room temperature. Fluorescently labelled samples were run on an Amnis^®^ ImageStream^®X^ Flow Cytometer (Luminex, Belgium) to assess antibody specificity and to define gates for subsequent analysis.

ImageStream^®X^ recovery rate experiments: To evaluate the recovery rate of the ImageStream^®X^ (Luminex, Belgium), duplicate experiments with cellular dilutions of 20, 100, 200 and 1000 paraformaldehyde-fixed colorectal cancer cells labelled with anti-CK-FITC (Miltenyi Biotec, Germany) were spiked in a constant number (5000) of carrier leukocytes from a healthy donor pre-labelled with anti-CD45-APC-Cy7. Cell counts were performed as previously described for IsoFlux^TM^ (Fluxion, Biosciences Inc., Alameda, CA, USA) control experiments but after fixation and labelling to avoid cell loss during washes. The efficiency of the ImageStream^®X^ (Luminex, Belgium) in detecting CTCs was calculated by the rate of CK+ for each dilution.

Combined protocol efficiency experiments: To evaluate the combined efficiency of the two platforms, duplicate experiments with 300 cells (either SW480 or HT-29) were spiked in 5 mL of peripheral blood. Cell counts were performed as previously described for IsoFlux^TM^ (Fluxion, Biosciences Inc., Alameda, CA, USA) control experiments. Then, the appropriate volume from the 1/100 dilution was added to the blood sample, which was then treated with lysis buffer prior to CTC enrichment on the IsoFlux^TM^ (Fluxion, Biosciences Inc., Alameda, CA, USA) and CTC characterisation on the ImageStream^®X^ (Luminex, Belgium), as described below, for the patient samples.

### 2.2. Validation Experiments with Early Colorectal Cancer Samples

This proof-of-concept study was approved by the Ethical Committee (code: CPI-2017-11-SAS-4B) at the University Hospital San Cecilio and University Hospital Virgen de las Nieves (Granada) and included 16 early colorectal cancer patients and 5 healthy donors who signed informed consent forms. Peripheral blood samples (5 mL) were extracted before (*n* = 16) and one month after surgery (*n* = 9) and processed in the Liquid Biopsy and Cancer Interception (LB&CI) laboratory at the GENyO centre within 4 h of extraction. Briefly, samples were lysed with 1× Red Blood Cell Lysis Solution (Miltenyi Biotech, Germany) following the manufacturer’s protocol. Then, the mononuclear cell pellet was washed with 1× PBS, centrifuged at 300× *g* for 10 min with moderate-speed deceleration and resuspended in 40 μL of pre-washed EpCAM-conjugated microbeads (Fluxion). Samples were incubated for 2 h at 4 °C in a roller rotator to facilitate binding with the microbeads. Then, after conditioning IsoFlux^TM^ (Fluxion, Biosciences, Inc., Alameda, CA, USA) cartridges with 1× Binding Buffer (Fluxion, Biosciences, Inc., Alameda, CA, USA) and priming, samples were loaded onto the cartridges for semi-automatic isolation of CTCs. Isolated CTCs were retrieved from the cartridge using 50 μL of 1× Binding Buffer and transferred to a Protein LoBind tube (Eppendorf, Germany). Treatment with 1 unit of papain (LK003176, Worthington, OH, USA) per sample for 20 min at 37 °C was performed before cell staining to release beads from cells, facilitating their subsequent characterisation. Cells were fixed and permeabilised using 1.7% paraformaldehyde and 0.2% Triton. Then, antibody labelling for cytokeratin (CK), BRAF^V600E^, PD-L1 nd CD45 was performed using the antibodies in Table 1 and 7-AAD (for nuclear staining) for 20 min at room temperature. Fluorescently labelled samples were run on an ImageStream^®X^ Flow Cytometer (Luminex, Belgium). The acquisition template was generated using the cell lines as controls, and laser intensities were as follows: 10 mW for 405 nm laser, 25 mW for 488 nm laser, 20 mW for 642 nm laser and 0.5 mW for 785 nm laser. Samples were acquired at 60× with a low flow rate using the Inspire^®^ software v 200.1.681.0 (accessed on 19 November 2021. Each file was analysed using the IDEAS^®^ software v 6.2 (accessed on 19 November 2021), as described in Section 3.2.

### 2.3. Statistical Analyses

Statistical analyses were performed using the SPSS v.25 software (accessed on 19 November 2021), and graphs were generated using GraphPad Prism v.8.0.2. (accessed on 19 November 2021) Student’s t-test was used when comparing two groups, and more than two groups were compared using one-way analysis of variance (ANOVA) followed by Tukey’s post hoc analysis. SPSS v.25 (accessed on 19 November 2021) was used for hierarchical clustering with the between-group linkage method and Pearson correlation analyses.

## 3. Results

### 3.1. Protocol Implementation for CTC Enrichment and Phenotypic Characterisation Combining the IsoFlux and ImageStream Platforms

**Figure 1:**
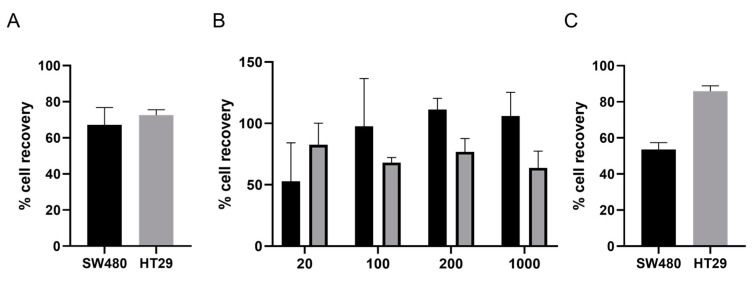
Efficiency assessment of a novel method for CTC isolation and phenotypic characterisation. (**A**) Efficiency of CTC enrichment for 300 colorectal cancer cell lines using the IsoFlux^TM^ platform. (**B**) Percentage of recovery of the ImageStream^®X^ platform using serial dilutions of cells from 20 to 1000 on the x-axis. (**C**) Combined efficiency of both platforms using spike-in experiments of 300 cells in peripheral blood of healthy donors. In all graphs, SW480 is shown in black, and HT-29 is shown in grey. Mean and standard deviation are shown for all experiments performed in duplicate.

Experiments using 300 cells in PBS were conducted in duplicate to assess the isolation efficiency of the IsoFlux^TM^ (Fluxion, Biosciences Inc., Alameda, CA, USA) system. Recovery rates of 72.5% and 67.2% were achieved for HT29 and SW480, respectively, as determined by visualising cytokeratin-positive cells under the fluorescent microscope (Figure 1A).

Subsequently, recovery rates and the limit of detection of the ImageStream^®X^ Flow Cytometer (Luminex, Belgium) were determined using a range of between 20 and 2000 cancer cells spiked in carrier leukocytes from healthy donors. Efficiencies varied between 50% and 100% and increased with increasing numbers of cells for SW480, whereas for HT29, efficiencies were 82.5%, 68.0%, 76.8% and 63.8% for 20, 100, 200 and 1000 cells, respectively, and did not change significantly with increasing cell numbers (Figure 1B).

Finally, efficiencies for the combination of the IsoFlux^TM^ (Fluxion, Biosciences Inc., Alameda, CA, USA) and ImageStream^®X^ Flow Cytometer (Luminex, Belgium) platforms were assessed using 300 spiked cancer cells in blood samples from healthy donors. Samples were treated in the same way as those from cancer patients and thus lysed for erythrocyte content, loaded onto the IsoFlux^TM^ (Fluxion, Biosciences Inc., Alameda, CA, USA) for CTC enrichment, stained with the antibody mix and run on the ImageStream^®X^ Flow Cytometer (Luminex, Belgium) for phenotypic characterisation. Experiments were carried out in duplicate, and overall efficiencies were 85.3% and 53.7% for HT29 and SW480, respectively (Figure 1C).

### 3.2. Analysis Protocol on the IDEAS Software 

We developed an analysis protocol on the IDEAS^®^ software (Luminex, Belgium) for the identification and phenotypic characterisation of CTCs isolated with the IsoFlux platform. A compensation matrix generated using compensation beads conjugated with each antibody was applied to all .rif files just before analysis. Then, a template for the analysis of all samples was generated using single controls (positive/negative) to locate gates for each cell population. Finally, all samples were analysed in batches using the same template, and statistical reports including the variables of interest were generated.

**Figure 2:**
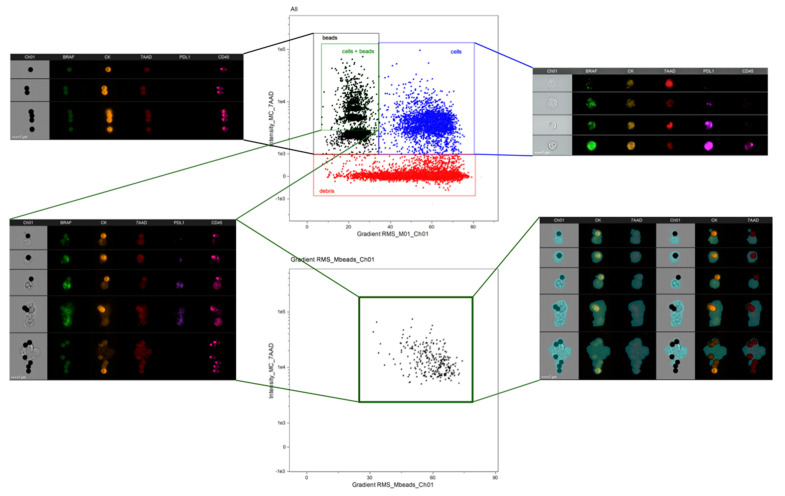
Optimisation of the analysis protocol for CTC characterisation using the IDEAS software. Top scatter plot shows all events acquired by the ImageStream^®X^ before applying the mask. Cells are represented by nuclear staining (7-AAD positivity) on the y-axis and Gradient_RMS_M01 feature (focus of the sample) on the x-axis. Bottom scatter plot shows the same population after applying a personalised mask for IsoFlux magnetic bead removal, improving the focus of CTCs attached to beads. Images at the edges are examples for each region.

Although we demonstrated that the magnetic beads used during CTC isolation are compatible with the ImageStream^®X^ (Luminex, Belgium) platform, they emit fluorescence at any laser intensity and thus need to be removed from the analysis; otherwise, fluorescence intensities will be overestimated. We treated the samples with papain to detach most of the magnetic beads from CTCs, facilitating their analysis. The use of papain increased the percentage of cytokeratin-positive bead-free cells from 46.9% to 91.38% (*p* = 0.024) (Figure S1A); however, some CTCs still had beads attached to their membrane (Figure 2, top). Thus, we ran the whole sample after papain treatment (both supernatant and pellet) to reduce the risk of losing CTCs. As shown in Figure 2, cells were identified using the “Gradient RMS_M01” feature and “Intensity_MC_7ADD”. Cells without magnetic beads were had “Gradient RMS_M01” values greater than 40 and nuclear intensities greater than 1000; however, cells attached to beads had “Gradient RMS_M01” values below 30 (this might be considered a non-focussed event).

As we wanted to remove fluorescence originating from the magnetic beads, we developed a mask defined as: [M01—LevelSet(M01, Ch01, Dim, 5)], which identifies the magnetic bead as a dense black circle on the brightfield image and removes it from the other channels, reducing the overestimation of fluorescence. As shown in Figure 2 (bottom), when plotting cells attached to beads after the application of this mask, not only did the fluorescence decrease, but “Gradient RMS_M01” also increased beyond 30, confirming the elimination of the beads from the analysis and improvement of the cell focus.

Subsequently, the combined populations of cells without beads and cells with beads after the application of our personalised mask were plotted according to the fluorescent intensities for each marker, allowing identification of CTCs as cytokeratin-positive cells (with an intensity threshold of 3000). In addition, some leukocytes were identified in cytokeratin-negative cells using a CD45 threshold of 2000 (Figure S1).

### 3.3. Isolation and Phenotypic Characterisation of CTCs from Early Colorectal Cancer Patients

Peripheral blood samples from 16 early colorectal cancer patients and 5 healthy donors (Table 2) were enriched in circulating tumour cells (CTCs) following our optimised protocol for the IsoFlux system (Fluxion) and phenotypically characterised on the ImageStream^®X^ (Luminex, Belgium) imaging flow cytometer after the application of our analysis method.

**Figure 3:**
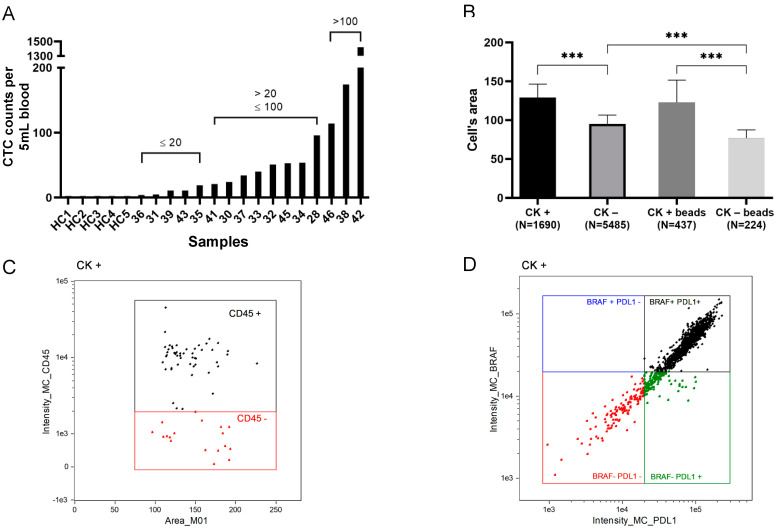
Circulating tumour cell (CTC) enumeration and characterisation based on cytokeratin (CK) expression. (**A**) The number of CTCs in 5 mL of peripheral blood (y-axis) per patient and healthy controls (HC) ordered by increasing cell counts (x-axis). Asterisk represents 5 healthy controls. (**B**) Mean size (in µm) comparison of cells depending on CK expression of either single cells (CK+/CK−) or CTCs attached to beads (CK+ beads/CK− beads). Number of cells used to calculate mean size is shown in brackets. (**C**) A scatter plot example of the CK+ population of a patient based on CD45 expression. (**D**) Characterisation of CTCs based on BRAF^V600E^ and PD-L1 expression. *** *p* value is <0.001 for ANOVA test followed by Turkey’s post hoc analyses.

CTCs were identified by their CK-positive expression in all patients and lack thereof in all healthy controls, although at different levels, with median CTC counts of 37 cells [IQ 13.0–85.5]. Among the 16 CRC patients, at baseline (before surgery), 31% (5/16) had ≤20 CTCs, 44% (7/16) had between 21 and 100 CTCs and 25% (4/16) had more than 100 CTCs (Figure 3A). Interestingly, the patient with the greatest number of CTCs (*n* = 1421) and the presence of 92 CTC clusters (Table 2) had a colonoscopy result showing high-grade dysplasia, whereas the remaining patients all had adenocarcinomas. Furthermore, we assessed the cell area and found that CTCs were significantly (*p* < 0.0001) larger than CK-negative cells independently of whether they were bound to beads (129.3 ± 36.6 and 94.7 ± 11.4, respectively) or not (121.1 ± 28.4 and 76.5 ± 28.1, respectively) (Figure 3B). The expression of CD45 in CK-negative cells confirmed that they were leukocytes (Supplementary Figure S1B), although we also identified high levels of CD45 expression on CK-positive cells for all patients. In particular, the mean CD45 expression on CTCs was 88.4% (range 57.1–100) (Figure 3C).

Both intra- and inter-individual phenotypic heterogeneity in BRAF^V600E^ and PD-L1 expression was identified within the population of CK-positive cells. Thus, we identified four CTC subpopulations with different frequencies among patients (Figure 3D and Table 2).

**Figure 4:**
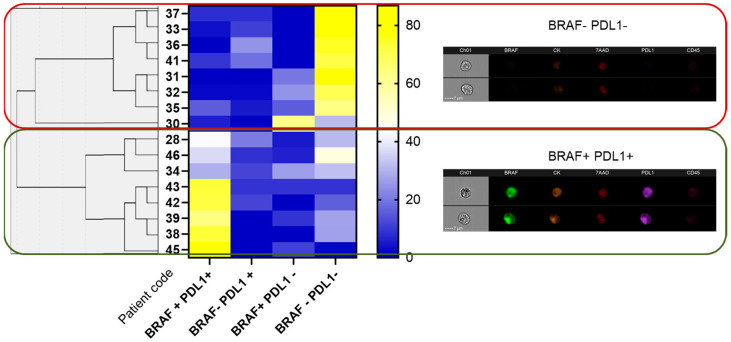
Characterisation of CTC subpopulations from CRC patients based on BRAF^V600E^ and PD-L1 expression. The left part of the figure shows a hierarchical cluster between groups, the middle part shows a heatmap representing percentages of either BRAF^V600E^ and/or PD-L1 expression and the right part shows an example of two CTCs belonging to each of the main subpopulations, either double-negative (BRAF^V600E^−/PD−L1−) or double-positive (BRAF^V600E^+/PD-L1+).

We identified that BRAF^V600E^ expression (measured as fluorescence intensity) was highly correlated with CK expression only in the BRAF+/PD-L1− population (*p* = 0.035), whereas PD-L1 expression was independent of either CK or BRAF^V600E^ in all four populations. We did not find a significant correlation (*p* = 0.079) between BRAF^V600E^+/PD-L1− and BRAF^V600E^−/PD-L1+ populations, suggesting that the expression of both markers in CTCs was independent. However, we did find a significant (*p* < 0.0001) negative correlation between the double-positive (BRAF^V600E^+/PD-L1+) and double-negative (BRAF^V600E^−/PD-L1−) populations, suggesting that they might serve as classification tools. In fact, the most frequent CTC subpopulation was the double-negative (100%), followed by the double-positive (88%). More variability was found in single-positive populations, which were found in 69% and 63% of the patients (for PD-L1+ and BRAF^V600E^+, respectively). Hierarchical clustering between groups identified two main clusters according to double positivity (BRAF^V600E^+/PD-L1+) and double negativity (BRAF^V600E^−/PD-L1−) (Figure 4). We also recognised a CK−/CD45+ population expressing PD-L1 in 62.5% (10/16) patients, whereas we only identified BRAF^V600E^ positivity in the CK−/CD45+ population in 18.75% (3/16).

Furthermore, we analysed PD-L1 localisation using the “Co-localization wizard analysis” (Figure S2), as it was previously suggested that nuclear staining was associated with poorer outcomes, and we identified 8/16 individuals with nuclear co-localisation of PD-L1 (Table 2).

**Figure 5:**
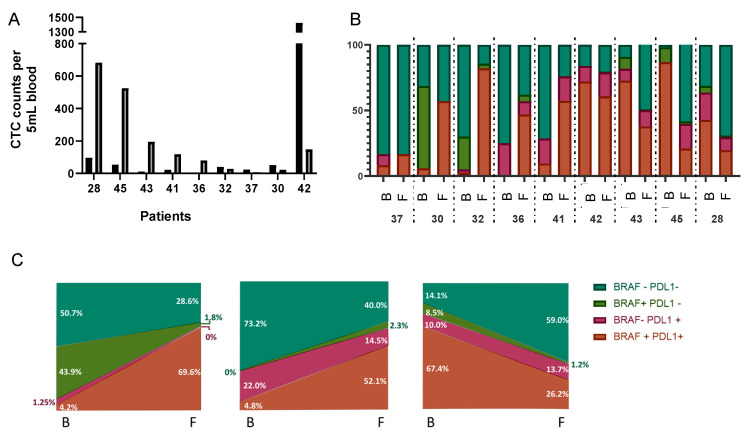
Evaluation of CTC counts and subpopulation evolution during patient follow-up. (**A**) Bar graph representing CTC numbers in 5 mL of peripheral blood at baseline (black) and one month after loco-regional surgery (grey) for 9 patients. (**B**) Evolution of the 4 CTC subpopulations based on BRAF^V600E^ and/or PD-L1 expression. For each patient, B indicates baseline, and F indicates follow-up. (**C**) Model of the CTC subpopulations’ main evolution processes. Colours are: brown: BRAF^V600E^+/PD-L1+; pink: BRAF^V600E^−/PD-L1+; green: BRAF^V600E^+/PD-L1−; and cyan: BRAF^V600E^−/PD-L1−.

Finally, we evaluated pre-surgery and post-surgery (1 month) samples in nine patients to demonstrate that our protocol is able to detect changes in CTC counts as well as the evolution of CTC subpopulations. The median number of CTCs increased to 117 cells [IQ 24.5–359.0] in the follow-ups. Comparisons between basal and follow-up CTC counts showed that 55.6% (5/9) of patients had an increase in CK+ CTCs after loco-regional surgery, whereas the number of CTCs decreased only in four patients (Figure 5A). Furthermore, the distribution among CTC subpopulations changed in all patients during the follow-up, although the relationship with the clinical and pathological characteristics of the patients needs further investigation (Figure 5B). Three main trends were observed: loss of BRAF+/PD-L1− and gain of BRAF+/PD-L1+ populations (Figure 5C, left), gain of BRAF+/PD-L1+ with loss of BRAF−/PD-L1− (Figure 5C, middle) and vice versa: gain of BRAF−/PD-L1− with loss of BRAF+/PD-L1+ (Figure 5C, right). CTCs from patients 30 and 32 behaved similarly in the follow-up, with loss of BRAF+/PD-L1− and gain of BRAF+/PD-L1+ populations, whereas CTCs from patients 36 and 41 gained BRAF+/PD-L1+ and lost BRAF−/PD-L1− populations. Finally, CTCs from patients 42, 43, 45 and 28 all gained the BRAF−/PD-L1− population and lost the BRAF+/PD-L1+ population. We were not able to analyse CTC subpopulation evolution over time for patient 37, as chemotherapy was applied before follow-up sampling. Thus, only six CTCs were detected 1 month after surgery, and changes among populations were not observed. Interestingly, this patient was the only one with recurrence and died from liver and lung metastases 5 months after loco-regional surgery.

## 4. Discussion

The clinical utility of identifying circulating tumour cells in colorectal cancer patients has already been described by several authors [15,22,23,25,27,38,39]. However, current protocols for the isolation and characterisation of CTCs are mainly limited to the microscopic evaluation of one or two markers, compromising the ability to identify different CTC subpopulations. Here, we developed a joint protocol for the enrichment and phenotypic characterisation of CTCs using the combination of a semi-automatic microfluidic-based platform (IsoFlux^TM^, Fluxion, Biosciences Inc., Alameda, CA, USA) and an imaging flow cytometry system (ImageStream^®^, Luminex, Belgium). We achieved a combined recovery efficiency of almost 70%; thus, our protocol increases the likelihood of detecting CTCs, as isolation efficiencies are higher than those of other methods [5,6]. Furthermore, phenotypic characterisation allowed the multi-parametric evaluation of markers, and CTC assessment was conducted automatically, reducing manual bias. 

Development of this protocol was accomplished after improvements of several limitations of the combination of the two technologies, mainly trying to reduce fluorescence. Although acquisition by the ImageStream^®X^ must be performed with laser intensities that do not saturate the fluorescence signal, we had some excess fluorescence due to: (a) bead autofluorescence, (b) out-of-focus events, (c) cellular autofluorescence, (d) clusters of cells and (e) cellular debris. The reduction in this fluorescent noise was achieved thanks to several improvements performed during the protocol optimisation. One of them was pre-treatment with papain, which reduced the number of beads attached to cells, allowing clearer characterisation of cells that would otherwise be masked by bead clumps. Furthermore, application of the personalised mask for bead removal improved the focus of cells bound to beads, allowing better characterisation and reducing the overestimation of fluorescence due to beads. Despite the automation of the analysis protocol, some manual characterisation was still needed, especially when accounting for cell clusters, as it is known that cell aggregates might be subject to non-specific labelling, producing false positives that need to be assessed manually by an expert eye.

We defined CTCs as cells expressing cytokeratin (either bound to IsoFlux^TM^ beads or not, as we pre-treated samples with papain to facilitate their analysis). Cut-off values of CTCs in non-metastatic colorectal cancer have been defined as between 1 and 5 cells in different studies [23,25,38,39]. However, as our methodology is more sensitive and efficient, we detected more CTCs per patient, and thus, cut-off values could be slightly elevated. 

Furthermore, CTCs should be negative for the common leukocyte marker CD45, as this is widely used to discriminate CTCs from other mononuclear peripheral cells. However, in our cohort, we identified many CK+ cells also expressing CD45 (88.4%). This has not previously been reported for colorectal cancer, but CTCs expressing EpCAM and CD45 were shown to be a poor prognostic marker in lung cancer [40], suggesting that we might be identifying a potentially aggressive population.

Our methodology for the semi-automatic isolation and multi-parametric characterisation of CTCs allowed patients to be classified into two major groups based on BRAF^V600E^ and PD-L1 expression. These two markers are usually evaluated in tumour tissue for treatment response assessment [33,41]. *BRAF* mutant tumours are associated with poorer prognosis [28]; in particular, the *BRAF^V600E^* mutation is associated with a lack of response to anti-EGFR therapies (such as cetuximab) if not treated simultaneously with an anti-BRAF^V600E^ treatment (such as encorafenib) [30]. However, the mutational status of *BRAF* on CTCs from CRC patients was only assessed using PCR, with the main purpose of evaluating its correlation with tissue mutational status [42]. On the other hand, the expression of PD-L1 is also considered an independent prognostic factor in patients with stages II/III [43]. The presence of PD-L1 on circulating tumour cells is known to mediate immune escape, and lung cancer patients with PD-L1+ CTCs before immunotherapy showed worse outcomes [44]. Thus, the evaluation of PD-L1 expression on CTCs, before treatment, in advanced solid tumours (including CRC) was shown to be a predictor of treatment response [45]. Furthermore, poorer survival was associated not only with PD-L1 expression on CTCs but also with its nuclear localisation [37]. Our novel protocol allowed automatic analysis of nuclear PD-L1 expression on CTCs, as we used the co-localisation feature for both fluorescent signals. This might be used as a tool to identify individuals with poorer outcomes.

A relationship between microsatellite instability, BRAF^V600E^ and PD-L1 expression was demonstrated in colorectal cancer tissues [46], suggesting that tumours with PD-L1 positivity are more aggressive. Here, we identified both markers in CTCs from early colorectal cancer patients at baseline and were able to classify them into two main groups: those with CTCs that were either positive or negative for both markers. Our data suggest that the combination of a highly efficient CTC isolation methodology together with a multi-parametric analysis using imaging flow cytometry allows identifying prognostic markers in early CRC patients. Furthermore, we were able to detect changes in the CTC subpopulation distribution during the follow-up of patients, identifying three main trends: either gain of PD-L1+ in the BRAF+/PD-L1− population, gain of the two markers in BRAF−/PD-L1− population or loss of the two markers. Our findings suggest that CTC characterisation using the ImageStream^®X^ might be used as a monitoring tool to evaluate the evolution of CTCs over time. Characterisation of CTCs using our protocol could be used in precision medicine as a stratification tool for identifying patients who are likely to respond to a specific treatment; however, our results need further validation in a larger cohort, and clinical evolution data need to be included to gain robustness and clinical utility.

## 5. Conclusions

The combination of the semi-automatic microfluidic-based platform (IsoFlux^TM^) and the imaging flow cytometry system (ImageStream^®X^) allows an improvement in the two most important phases of circulating tumour cell assessment: enrichment and phenotypic characterisation. Optimisation of this novel protocol allowed characterisation of CTCs in early colorectal cancer patients, enabling the identification of up to four different subpopulations based on BRAF^V600E^ and PD-L1 expression on cytokeratin-positive CTCs. Our results highlight the importance of evaluating multiple parameters, such as size, expression levels or protein localisation, to identify intra- and inter-individual tumour heterogeneity.

## Figures and Tables

**Table 1:** List of antibodies used for phenotypic characterisation of circulating tumour cells on the ImageStream^®X^ platform.

Primary	Secondary	Fluorophore	Dilution	Reference	Brand	IS Ch
* Mouse IgG2a anti-cytokeratin 7/8	None	FITC	1/100	130-060-301	Miltenyi, Germany	2
Rabbit monoclonal [K21-F] to BRAF (mutatedV600E)	None	FITC	1/200	ab175637	Abcam, UK	2
Mouse monoclonal [CAM5.2] anti-human cytokeratin (7/8)	None	PE-CF594	1/100	563615	BD, USA	4
None	None	7-AAD	1/100	00-6993-50	Invitrogen, USA	5
Rabbit polyclonal to PD-L1	Goat anti-rabbit IgG	DyLight 405	1/100 1/100	PA5-28115 35551	Thermo Fisher, USA	7
Mouse monoclonal [2D1] anti-human CD45 APC CY7	None	APC-Cy7	1/100	557833	BD, USA	12

* Anti-CK-FITC was only used for control experiments to ensure epithelial origin, but for CTC characterisation, anti-CK-PE-CF594 antibody was used instead; abbreviation “IS Ch” refers to “ImageStream^®X^ channel”.

**Table 2:** Summary of early CRC patients analysed for CTC counts and CTC subpopulations. N indicates the number of circulating tumour cells (CTCs) or CTC clusters per 5 mL of peripheral blood. Abbreviations are: Y, yes; N, no; N/A, not applicable; and HC, healthy control.

Patient Number	CK+ CTCs (N)	CTC Clusters (N)	BRAF^V600E^+ PD-L1+ (%)	BRAF^V600E^− PD-L1+ (%)	BRAF^V600E^+ PD-L1− (%)	BRAF^V600E^− PD-L1− (%)	PD-L1 Nuclear Location
HC 1	0	0	0	0	0	0	N/A
HC 2	0	0	0	0	0	0	N/A
HC 3	0	0	0	0	0	0	N/A
HC 4	0	0	0	0	0	0	N/A
HC 5	0	0	0	0	0	0	N/A
37	24	0	8.3	8.3	0.0	83.3	Y
33	54	1	3.7	11.1	0.0	85.2	Y
36	4	0	0.0	25.0	0.0	75.0	N
41	21	0	9.5	19.1	0.0	71.4	N
31	5	0	0.0	0.0	20.0	80.0	N
32	40	0	2.5	2.5	25.0	70.0	N
35	19	1	15.8	5.3	15.8	63.2	N
30	51	0	5.9	0.0	62.8	31.4	N
28	96	0	42.7	20.8	5.2	31.3	N
46	114	0	36.8	8.8	6.1	48.3	Y
34	34	0	29.4	11.8	26.5	32.4	Y
43	11	1	72.7	9.1	9.1	9.1	Y
42	1421	92	72.1	11.8	0.0	16.1	Y
39	11	0	63.6	0.0	9.1	27.3	N
38	174	7	73.0	0.0	0.0	27.0	Y
45	53	0	86.8	0.0	11.3	1.9	Y

## Data Availability

Data supporting the reported results might be obtained from the authors upon request.

## Supplementary Materials

The following are available online at https://www.mdpi.com/article/10.3390/cancers13246386/s1, Figure S1: Protocol optimisation, Figure S2: Nuclear localisation of PD-L1 expression in CTCs.
