# Supplementary material for: Deep Phenotypic Characterisation of CTCs by Combination of Microfluidic Isolation (IsoFlux) and Imaging Flow Cytometry (ImageStream)
Source: Cancers (Basel). 2021 Dec 20;13(24):6386. doi: 10.3390/cancers13246386 (PMC8699219; doi:10.3390/cancers13246386)
Supplement: Supplementary file 1 [file cancers-13-06386-s001.zip › cancers-1494254-supplementary.pdf]

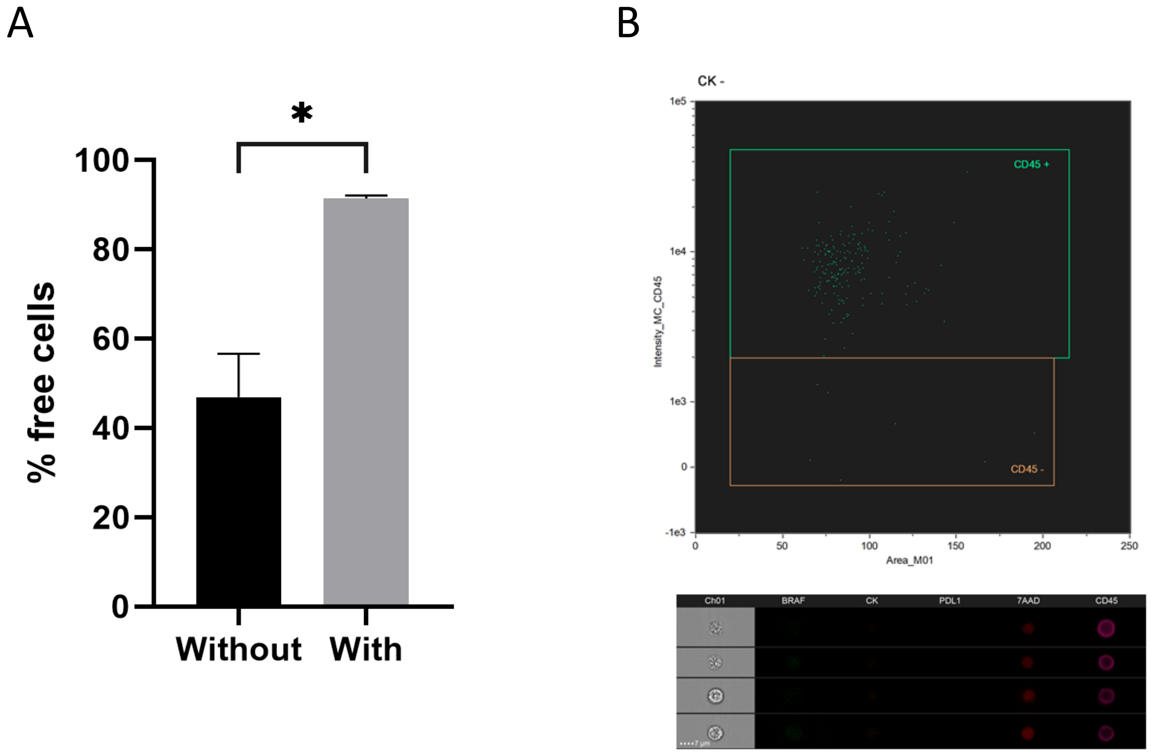

Supplementary figure S1: Protocol optimization. A is a bar graph showing the percentage of cytokeratin-positive beads-free cells without using papain (black) or adding papain (grey) for detachment of cells from beads. **B** is a scatter plot showing leukocytes by their CD45+ staining in a cytokeratin (CK) negative population. P value is X

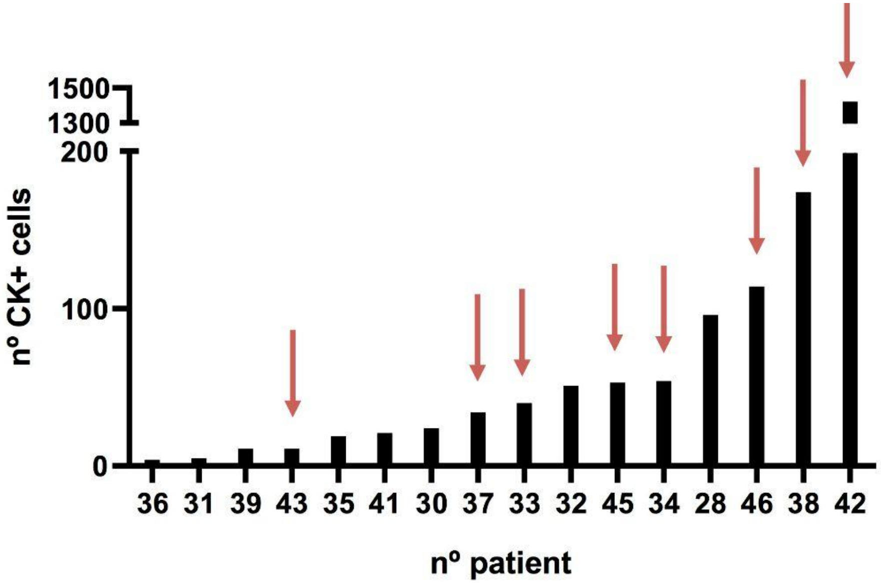

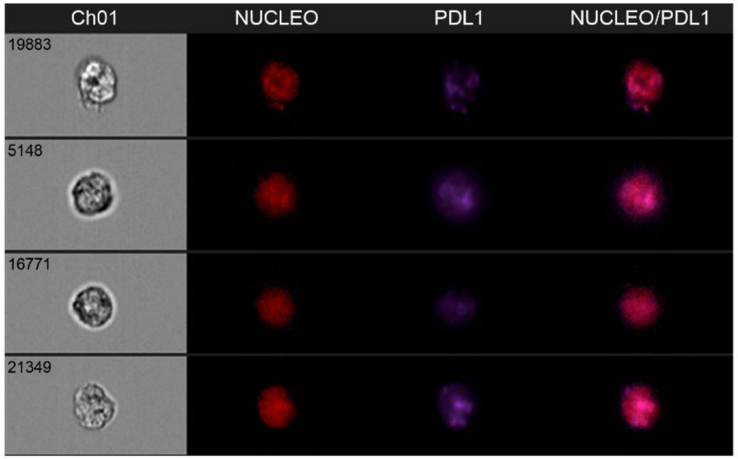

Supplementary figure S2: Nuclear localisation of PD-L1 expression on CTCs. Left part shows a bar graph with increasing number of cytokeratin (CK) circulating tumor cells. Patients with nuclear expression of PD-L1 is indicated with a red arrow. Right part shows an example of co-localised nuclear and PD-L1 staining.
